# Homogenous Population Genetic Structure of the Non-Native Raccoon Dog (*Nyctereutes procyonoides*) in Europe as a Result of Rapid Population Expansion

**DOI:** 10.1371/journal.pone.0153098

**Published:** 2016-04-11

**Authors:** Frank Drygala, Nikolay Korablev, Hermann Ansorge, Joerns Fickel, Marja Isomursu, Morten Elmeros, Rafał Kowalczyk, Laima Baltrunaite, Linas Balciauskas, Urmas Saarma, Christoph Schulze, Peter Borkenhagen, Alain C. Frantz

**Affiliations:** 1 Musée National d'Histoire Naturelle, Luxembourg; 2 Velikie Luki State Agricultural Academy, Velikie Luki, Russia; 3 Senckenberg Museum für Naturkunde Görlitz, Görlitz, Germany; 4 Leibniz-Institute for Zoo and Wildlife Research (IZW), Berlin, Germany; 5 Potsdam University, Institute for Biochemistry and Biology, Potsdam, Germany; 6 Finnish Food Safety Authority, Production animal and wildlife research unit, Oulu, Finland; 7 Department of Bioscience, Aarhus University, Rønde, Denmark; 8 Mammal Research Institute, Polish Academy of Sciences, Białowieża, Poland; 9 Nature Research Centre, Institute of Ecology, Vilnius, Lithuania; 10 University of Tartu, Department of Zoology, Tartu, Estonia; 11 Landeslabor Berlin-Brandenburg, Frankfurt (Oder), Germany; 12 Faunistisch-Ökologischen Arbeitsgemeinschaft S-H, Kiel University, Kiel, Germany; BiK-F Biodiversity and Climate Research Center, GERMANY

## Abstract

The extent of gene flow during the range expansion of non-native species influences the amount of genetic diversity retained in expanding populations. Here, we analyse the population genetic structure of the raccoon dog (*Nyctereutes procyonoides*) in north-eastern and central Europe. This invasive species is of management concern because it is highly susceptible to fox rabies and an important secondary host of the virus. We hypothesized that the large number of introduced animals and the species’ dispersal capabilities led to high population connectivity and maintenance of genetic diversity throughout the invaded range. We genotyped 332 tissue samples from seven European countries using 16 microsatellite loci. Different algorithms identified three genetic clusters corresponding to Finland, Denmark and a large ‘central’ population that reached from introduction areas in western Russia to northern Germany. Cluster assignments provided evidence of long-distance dispersal. The results of an Approximate Bayesian Computation analysis supported a scenario of equal effective population sizes among different pre-defined populations in the large central cluster. Our results are in line with strong gene flow and secondary admixture between neighbouring demes leading to reduced genetic structuring, probably a result of its fairly rapid population expansion after introduction. The results presented here are remarkable in the sense that we identified a homogenous genetic cluster inhabiting an area stretching over more than 1500km. They are also relevant for disease management, as in the event of a significant rabies outbreak, there is a great risk of a rapid virus spread among raccoon dog populations.

## Introduction

Non-native species pose a great threat to the integrity of natural systems and are of evolutionary interest because genetic processes may play a role in their establishment and spread. Because genetic founder effects can be overcome by high propagule pressure, a positive association between introduction effort and invasion success has been reported [1–3]. The extent of gene flow during the range expansion of an invader does, however, also influence the amount of genetic diversity retained in newly founded populations [4]. Genetic diversity at the expanding range front may be declining as a result of recurrent bottlenecks and founder effects [5], which, in the absence of admixture, can furthermore lead to significant gradients in allele frequencies among populations [6,7]. Alternatively, a large amount of migration and gene flow between neighbouring populations will preserve the genetic diversity of the source population [4]. Given the outcomes of these alternative scenarios, genetic analyses offer a promising tool to understand the colonisation history of invading species.

The raccoon dog (*Nyctereutes procyonoides*) is a canid with a high reproductive rate, short generation times, high population turnover and a generalised diet [8–10]. While it is native to Eastern Asia, more than 9.000 animals originating from the Russian Far East were released during several introduction events in the western part of the former Soviet Union between the 1930s and the 1950s. Animals were first bred in fur farms and then intentionally released into the wild [9, 11]. Some populations in Belarus and the Ukraine were founded by animals that had been captured in the introduction areas [12]. Many introductions were successful and the populations started to spread at a rate of 40 km per year, with some individuals dispersing up to 500 km from the introduction sites within three years after their release [9, 11,13,14]. Today, the raccoon dog is widespread in Northern and Eastern Europe and still spreading in Central Europe (e.g. [9, 15]).

The raccoon dog is of special management concern because it can act as a vector for several diseases and parasites of humans, domestic animals and wildlife [9,16,17]. Importantly, the species is highly susceptible to rabies and considered to be a secondary host of high importance, particularly in north-eastern Europe [16, 18]. In the (non-negligible) event of another rabies outbreak [19], the virus maybe further spread by dispersal of young raccoon dogs [20]. In order to adapt rabies management plans [18], it is important to use genetic techniques to understand the frequency of the species’ long-distance dispersal and, more generally, the connectivity of raccoon dog populations. Finally, parasites evolve quicker than hosts [21] and genetically depauperated hosts [22] or populations [23] may be more susceptible to pathogens and parasites than genetically more diverse ones [24]. It would therefore also be important to assess the influence of the range expansion on the retention of nuclear genetic diversity of the raccoon dog (see also [25]).

Here, we analyse the population genetic structure of the raccoon dog in large parts of its north-eastern and Central European range in order to reconstruct the species’ colonisation patterns. We hypothesized that high propagule pressure and a high number of migrants and thus gene flow (including frequent long distance dispersal events) resulted in high genetic diversity and population connectivity throughout the invaded range. By gaining a better understanding of inter-population connectivity of our model species, we may contribute to the development of more effective, data-informed management and control programs.

## Material and Methods

**Fig 1:**
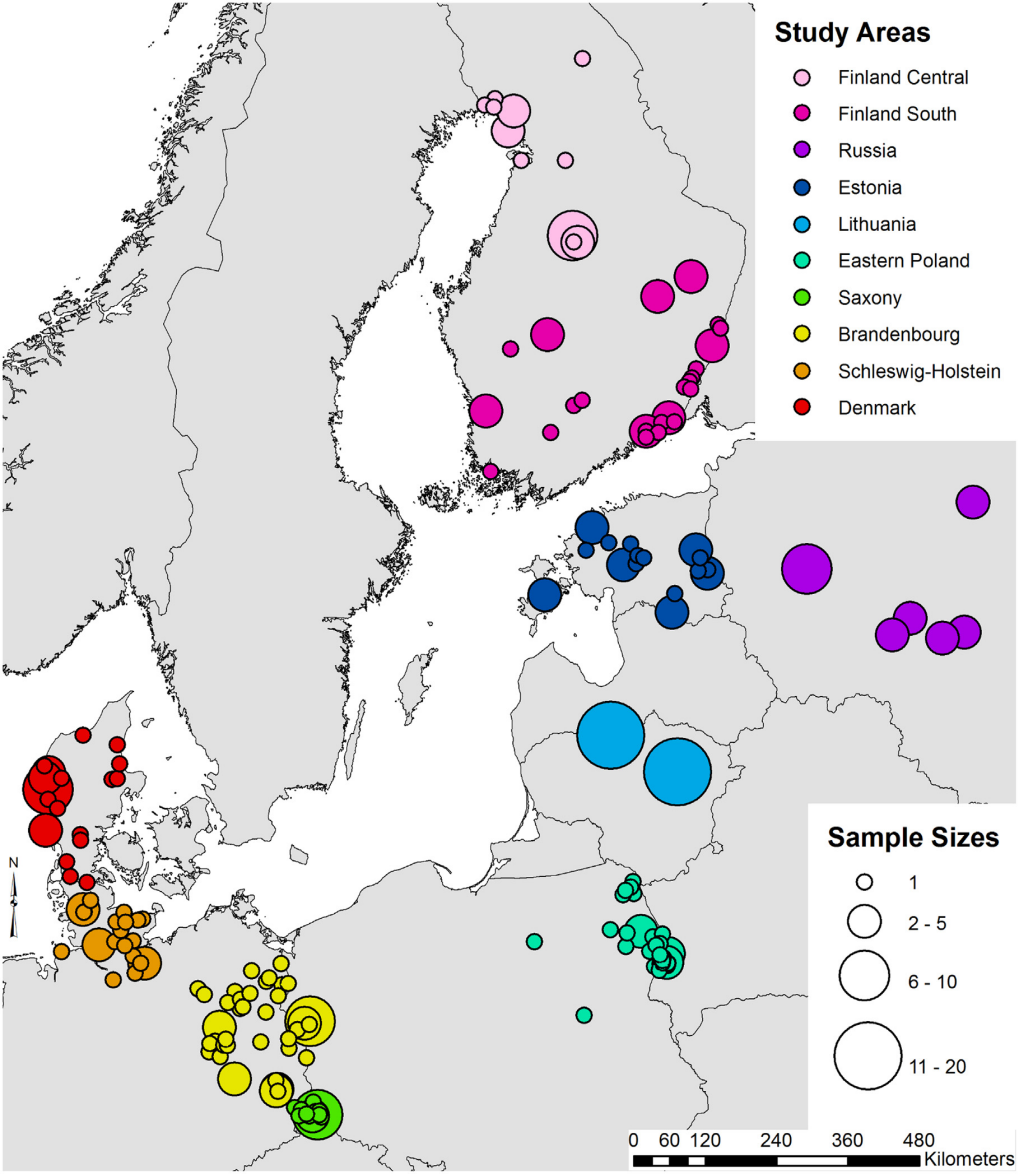
Sample distribution and location of the pre-defined study populations. The size of a circle is representative of the number of samples collected from that locality.

We collected tissue samples (muscle or ear) from a total of 332 hunted or road-killed raccoon dogs originating from seven European countries (Fig 1). Throughout the study area no special permits (other than a general hunting licence) were required to legally hunt raccoon dogs or collected road-killed individuals. No author was involved in hunting and no animal was killed with the aim of providing samples for this study. All authors obtained samples (including road-kill) directly from licenced hunters and, in one case, from the Danish Nature Agency (the general Danish wildlife management authority). The following authorities issue the relevant general hunting licences: Denmark, Danish Nature Agency; Estonia, Estonian Environmental Agency; Finland, Finnish Wildlife Agency; Germany, Lower Hunting Authorities; Lithuania: Utenos and Siauliai Regional Environmental Protection Departments; Poland, State Forest Administrations Augustów, Białowieża, Browsk, Giżycko, Hajnówka, Jedwabno, Płaska, Pomorze and Waliły; Russia, State Committee of the Pskov region on natural resources and environmental protection & The Ministry of Natural Resources and Ecology of the Tver region.

In order to allow basic population genetic analyses, we pre-defined ten populations (Fig 1): the German samples were sub-divided according to Federal State of origin and, due to the large geographic sampling area, the Finnish samples were subdivided into a southern and central population. Polish raccoon dogs that were sampled close to the German border (n = 9) were included in the population of the German Federal State of Brandenburg.

Tissues samples were stored in absolute ethanol or at -20°C until extraction. DNA was extracted using an ammonium acetate-based salting-out procedure [26]. Raccoon dogs were genotyped at 16 microsatellite loci: *DGN14*, *FH2174*, *FH2226*, *FH2281*, *FH2289*, *FH2316*, *FH2541*, *FH2658*, *Ren161A12*, *Ren162B09*, *V142*, *V374*, *V402*, *V468*, *V602* [27] and *FH2097* [28] in four multiplex reactions (S1 Table). We amplified each multiplex using the Qiagen multiplex Kit (Qiagen, Hilden, Germany). Each multiplex reaction contained 1x Qiagen multiplex master mix, with primer concentrations varying between 0.1–0.3 μM (S1 Table). After drying 1 μl of DNA (10–30 ng/μl) for 15 min at 56°C in a 384-well PCR plate (Greiner Bio-One, Stonehouse, UK), multiplex reactions were performed under mineral oil in a total volume of 2 μl. Reactions started with a 15 min denaturation period at 95°C, followed by denaturation at 94°C for 30 s, annealing at 62°C for 90 s and extension at 72°C for 1min. Final incubation was at 68°C for 30 min. PCR products were genotyped using a capillary sequencer (ABI 3730XL, Applied Biosystems). Allele sizes were estimated using GENEMAPPER version 3.7 (Applied Biosystems). The genetic profiles of all samples consisted of at least 14 loci.

We used the ten pre-defined population to test if allele distribution at the microsatellite loci deviate from Hardy–Weinberg equilibrium (HWE) using the Markov chain method implemented in GENEPOP 3.4 [29], with 10,000 dememorisation steps, 500 batches and 10,000 subsequent iterations. The populations were tested for allele linkage disequilibria among loci using an exact test based on a Markov chain method as also implemented in GENEPOP 3.4. The false discovery rate technique was used to eliminate false assignment of significance by chance [30]. Given that the microsatellite loci used in this study were first identified in either the domestic dog (*Canis familiaris*) or the fox (*Vulpes vulpes*), we estimated null allele frequencies for each locus and population based on the Expectation Maximization (EM) algorithm [31] implemented in program FreeNA [32].

We used STRUCTURE v2.3.1 [33] to estimate *K*, the likely number of genetic clusters (= subpopulations). Ten independent runs for *K* = 1–10 were carried out with 10^6^ Markov chain Monte Carlo (MCMC) iterations after a burn-in period of 10^5^ iterations, using the model with correlated allele frequencies and assuming admixture. ALPHA, the Dirichlet parameter for the degree of admixture, was allowed to vary between populations. After deciding on the most probable number of subpopulations based on the log-likelihood values (and their convergence) associated with each *K*, as well as on the Δ*K* method by Evanno et al. [34], we calculated each individual’s percentage of membership (*q*), averaging *q* over ten runs. Secondly, we also analysed the data using the spatially explicit genetic clustering method that is implemented in the program BAPS v.6.0 [35]. In addition to the genetic data, the algorithm considers the specific geographic coordinates of each individual and modally assigns each individual to its population of origin. We performed ten runs for each of *K* = 2–10. Bar plots of assignments were generated using DISTRUCT 1.1 [36]. We also used GENETIX v.4.05.2 [37] to perform a factorial correspondence analysis (FCA) to visualise the genetic distance between the 10 predefined raccoon dog populations.

The level of genetic differentiation between the inferred clusters was quantified using *F*_ST_ [38] in SPAGeDi 1.4 [39] and significance was tested with 10,000 permutations of individual genotypes between populations. The relationship between genetic and geographical distances was examined to assess isolation-by-distance (IBD; [40]). We calculated the regression of *F*_ST_/(1-*F*_ST_) estimates for pairs of the ten pre-defined populations on the logarithm of the geographic distance between them using SPAGeDi 1.4. We used the average longitude and latitude of the individual samples as geographic coordinate for each pre-defined population. Given that the samples were collected from sites around the Baltic Sea (Fig 1), straight line-distances might not adequately describe the geographic distance separating the sampling populations. We therefore used ArcMap 10.3 (ESRI, Redlands, USA) to generate a resistance surface (based on a 30x30m grid) of the study area that gave a high cost value to water bodies. We then used the Landscape Genetics Toolbox [41] to calculate the length of the least-cost paths between the pre-defined populations (S1 Fig). These were then introduced as geographic distance in the regression analysis. The slope was tested for a significant difference from zero by 10,000 permutations of locations of individuals.

We estimated average number of alleles/locus as well as observed (*H*_obs_) and unbiased expected (*H*_eu_) heterozygosities [42] using GENETIX 4.05.2 [37]. Allelic richness (*A*_r_) was calculated using FSTAT v. 2.9.3.2 [43]. We estimated effective population sizes (*N*_e_) using the linkage disequilibrium method in program NeEstimator v.2.01 [44], estimating 95% confidence intervals using jack knifing and excluding rare alleles with frequencies less than 0.02. It has been suggested that this approach, which is based on the rationale that genetic drift will create non-random allele combinations in small populations with few parent individuals, is reliable if effective population sizes are not much larger than ca. 200 and the data set is based on 10 or more loci and population sample sizes of 25 or more [45]. These summary statistics were calculated both for the ten pre-defined populations as well as for the inferred genetic clusters. We used GenAlEx v.6.501 [46] to estimate the number of private alleles in each of the inferred genetic clusters.

In order to further reconstruct and confirm the pattern of the raccoon dog’s colonisation of central Europe, we compared competing scenarios (S2 Fig) regarding population history using Approximate Bayesian Computation (ABC) implemented in the program DIYABC 2.1.0 [47]. This software produces estimates of the relative likelihood of alternative scenarios in a coalescent framework. Focussing on central Europe, we wanted to differentiate between a stepping stone scenario, where newly created populations act as source for subsequent spread, and a scenario of considerable gene flow among populations. The former scenario is expected to lead to reduced effective population size and genetic diversity in subsequently founded populations [48–50], while the latter one is not expected to do so. Given that the animals were initially released in the western part of the former Soviet Union, we pooled the animals from Russia, Estonia and Lithuania into one source population. Given their close geographic proximity, the Brandenburg and Saxony samples were also pooled. All the four simulated, alternative population histories (and the priors for the time parameters) were set in such a way that the first population to split from the source population was Poland, Brandenburg/Saxony then split from Poland, then Schleswig-Holstein from Brandenburg/Saxony and finally Denmark from Schleswig-Holstein. We did not include Finland because it only can split from the source population and therefore has no relevance for colonisation pattern in Central-Europe. In scenario 1, all newly founded populations had the same effective population size. In scenario 2, all newly founded populations had the same effective population size, with the exception of Denmark, whose effective populations was set to be lower. In scenario 3, the Polish population had the same effective population size as the source population, the two German populations had the same effective population size that was lower compared with the source population and, finally, the Danish raccoon dogs had the lowest effective population size. In scenario 4, the effective population size of all five populations was different and decreasing from east to west. We used uniform priors for all parameters. All time parameters were set to a minimum of 10 and a maximum of 200, while the effective population size parameters were set to a minimum of 10 and a maximum of 2000, except the one of the source population, whose maximum was set to 5000.

Simulated data sets were created by requesting nine summary statistics (per population and per pairs of populations), including the number of alleles, genetic diversity, *F*_ST_ and δμ^2^ (genetic distance) pair wise divergence statistics. One million simulated data sets per scenario were generated to estimate posterior distributions. Each scenario was considered equally probable and reliability of scenarios was visualised through principal component analysis, whereas posterior probabilities of scenarios were compared by means of logistic regression. Parameters of the most likely scenarios were estimated by using the 10000 data closest to the observed data, applying a logit transformation to the parameters and choosing the mode of the posterior distribution as point estimate.

## Results

Locus *FH2174* deviated from HWE in nine of the ten pre-defined populations before correcting for multiple tests and in six populations after the correction (S2 Table), while no other locus exhibited a systematic heterozygote excess or deficiency. No pairs of loci were in linkage disequilibrium after correction for multiple tests. The null allele frequencies for locus *FH2174* ranged between 0.096 and 0.280. Apart from this locus, null allele frequencies were low, however, with only one locus having null allele frequencies >0.10 in any of the ten pre-defined populations (S2 Table). Only locus *FH2174* was thus excluded from all further analyses, which were consequently based on 15 loci.

**Fig 2:**
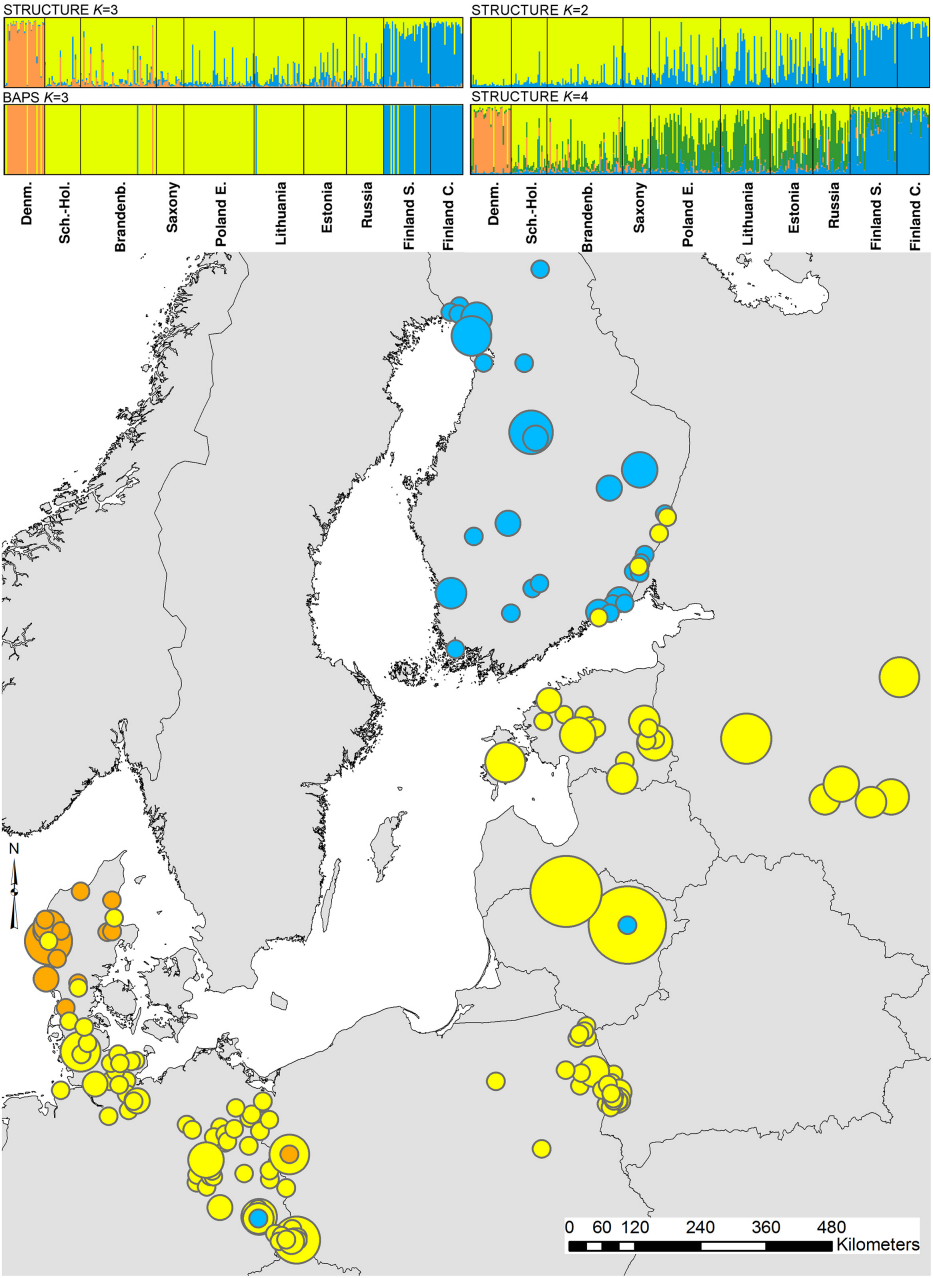
Geographic distribution of the BAPS derived clusters (*K* = 3), obtained using the spatial model. Different colours represent the model assignment of individuals to the different clusters. The size of the symbol is representative of the number of individuals sampled at that location (see also Fig 1). Inset: Summary of the assignment results obtained with STRUCTURE (*K* = 2 to 4) and BAPS (*K* = 3). Each individual is represented by a single vertical line, representing the individual`s estimated proportion of membership to the genetic cluster. Colours correspond to the clusters in the main figure. The order of the individuals was the same in both assignments. The STRUCTURE *K* = 4 bar chart represents results from the nine (out of ten) independent runs that converged on the same clustering solution.

**Fig 3:**
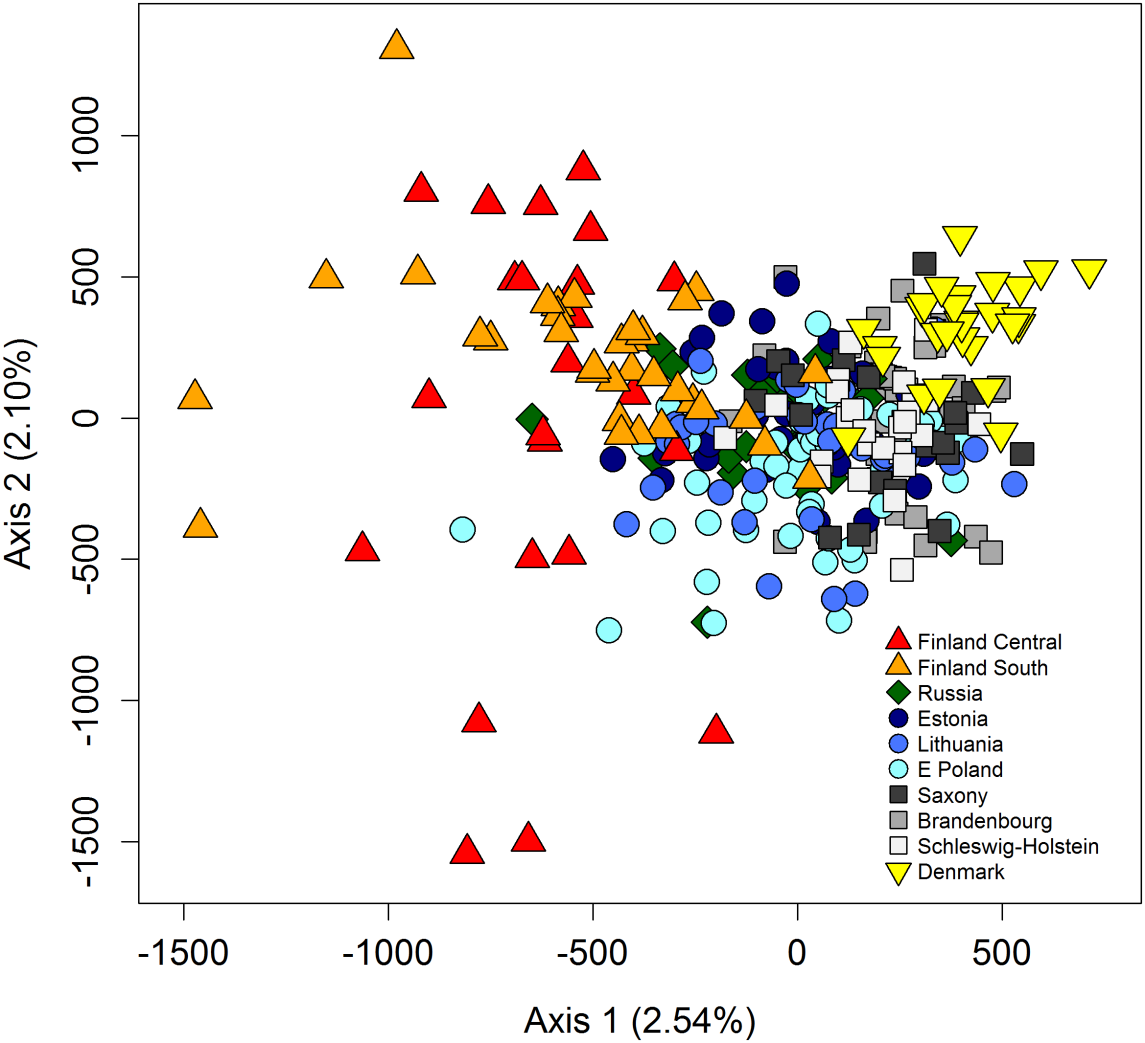
Factorial correspondence analysis of raccoon dogs from the 10 pre-defined European populations. The analysis was based on 15 microsatellite loci. The percentage of the total variation explained by each of the two axes is given.

The log-likelihood values and the Δ*K* method of the STRUCTURE analysis both suggested the presence of three genetic clusters in the data set (S3 Fig). Also, the spatially explicit clustering method in BAPS gave a probability of >0.99 for the presence of three genetic clusters. The three genetic populations identified by both algorithms corresponded in essence to Finland (the predefined populations Finland-central & Finland-south), Denmark and one larger cluster (henceforth referred to as ‘central cluster’) encompassing all the remaining pre-defined populations (Fig 2). The FCA confirmed the genetic distinctness of both the Finnish and the Danish population, while the German samples overlapped with the Eastern European samples (Fig 3). The STRUCTURE assignment results for *K* = 2 and *K* = 4 are also given in Fig 2. At *K* = 4, which had lower log-likelihood values than *K* = 3 (S3 Fig), STRUCTURE hints at the existence of a weak partition between individuals in the western central (German) and eastern central cluster.

The genetic differentiation between all three pairs of (BAPS-defined) clusters was significant, with Denmark and Finland exhibiting the highest level (*F*_ST_ = 0.071, *P*<0.001), followed by Denmark and the central cluster (*F*_ST_ = 0.041, *P*<0.001) and the central cluster and Finland (*F*_ST_ = 0.028, *P*<0.001). We found evidence for long-distance dispersal: both STRUCTURE and BAPS identified individuals there were sampled in the geographic distribution area of one cluster but genetically assigned to another one (Fig 2). While both algorithms often agreed on the identity of these dispersers, especially in relation to animals that were sampled in southern Finland and Denmark, this was not always the case. Finally, the position of individuals in the FCA also supports the presence of long-distance dispersal (Fig 3).

**Fig 4:**
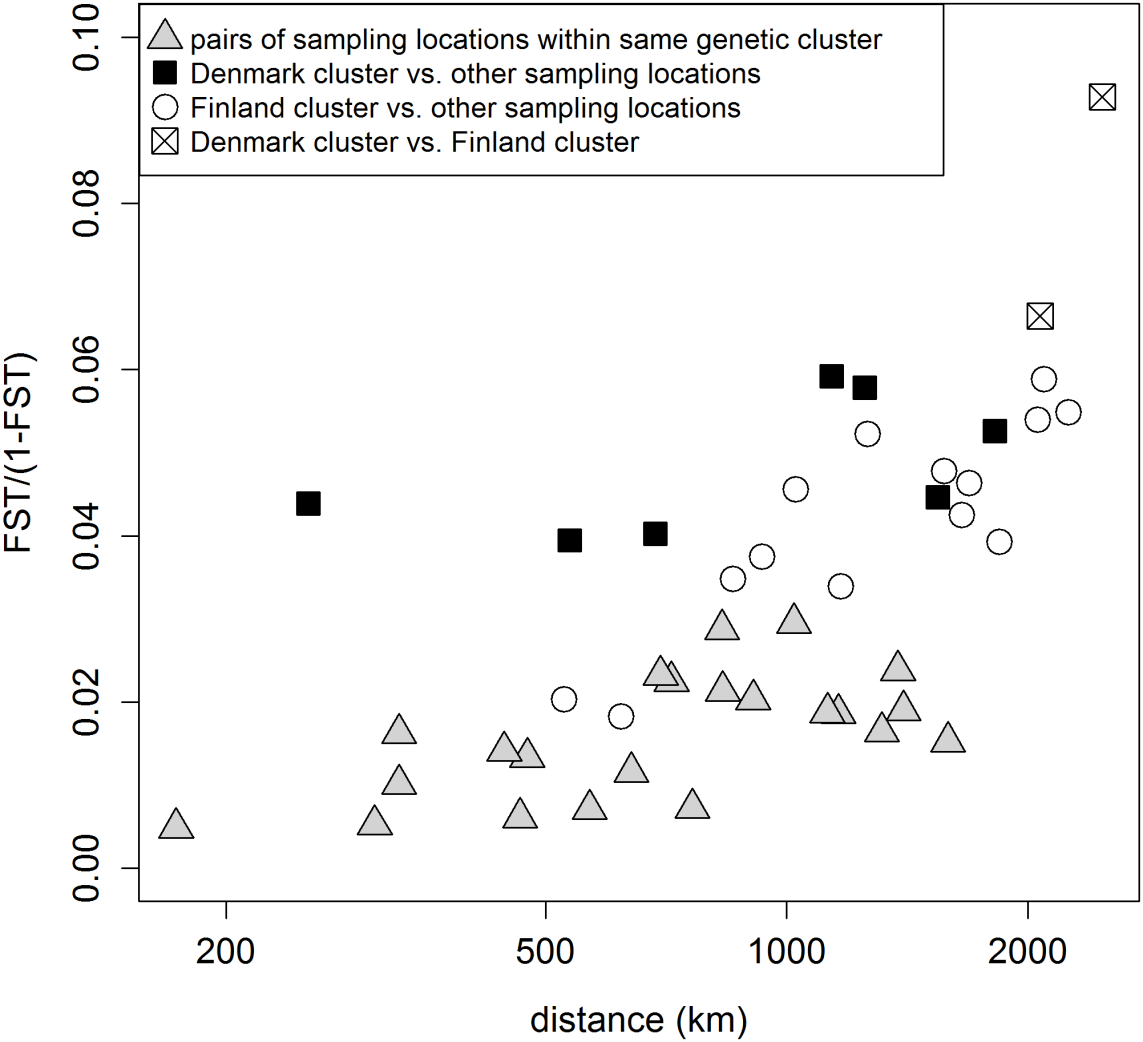
Genetic and geographic distance for pairs of sampled pre-defined populations. Genetic differentiation is given as *F*_ST_/(1-*F*_ST_), whereas the geographic distance was log-transformed. Geographic distances are given as effective geographic distances that only consider the shortest overland route between sampling points.

The whole dataset was characterised by a significant population-based IBD pattern (*b* = 0.021; *P*<0.001). However, the genetic differentiation between Denmark and each of the other pre-defined populations was larger than between pre-defined populations from the same genetic cluster that had similar pair wise geographic distances (Fig 4). While the pattern was less pronounced, the same applied to the two pre-defined populations from Finland. The pronounced overall IBD pattern therefore resulted from the presence of genetic discontinuities and the relative location of the pre-defined populations, rather than a genuine isolation-by-distance pattern. This is consistent with the lack of statistically significant IBD pattern (*b* = 0.008; *P* = 0.055) obtained when only considering the pre-defined populations in the large, central cluster.

Our results suggested that the Denmark cluster was genetically less diverse than the other two clusters, particularly in terms of number of alleles (Table 1) and that it had a smaller effective population size (Table 1 and S3 Table). Furthermore, there appeared to be a decrease in the number of alleles per population from east to west and to the north (S3 Table). In the FCA, the German samples only overlapped with a part of the eastern European cluster, also hinting at a reduced genetic diversity in western central Europe. The Finnish cluster was characterised by six alleles (at four loci) that did not occur in either of the two remaining clusters, while the large central cluster had 36 private alleles. The Danish cluster had no private alleles when considering the whole dataset, yet it did have one allele that did not occur in the central cluster and five that did not occur in the Finnish cluster.

**Table 1:** Genetic characteristics of the three inferred genetic clusters.

BAPS-defined cluster	Sample size	Microsatellite diversity				*N*_e_	95% CI
		*A*	*A*_R_	*Ho*	*He*_u_		
Denmark	25	4.7	4.6	0.664	0.607	22.7	15.4–34.9
Central Europe	252	9.7	6.8	0.688	0.709	513.1	334.1–991.6
Finland	55	7.4	6.4	0.668	0.685	87.4	61.7–139.3

*A*: no. of alleles; *A*_R_: allelic richness (minimum sample size of 22 diploid individuals); *Ho*: observed heterozygosity; *He*_u_: unbiased expected heterozygosity; *N*_e_: effective population size based on the linkage disequilibrium method; 95% CI: lower and upper 95% confidence intervals of *N*_e_ estimate

**Fig 5:**
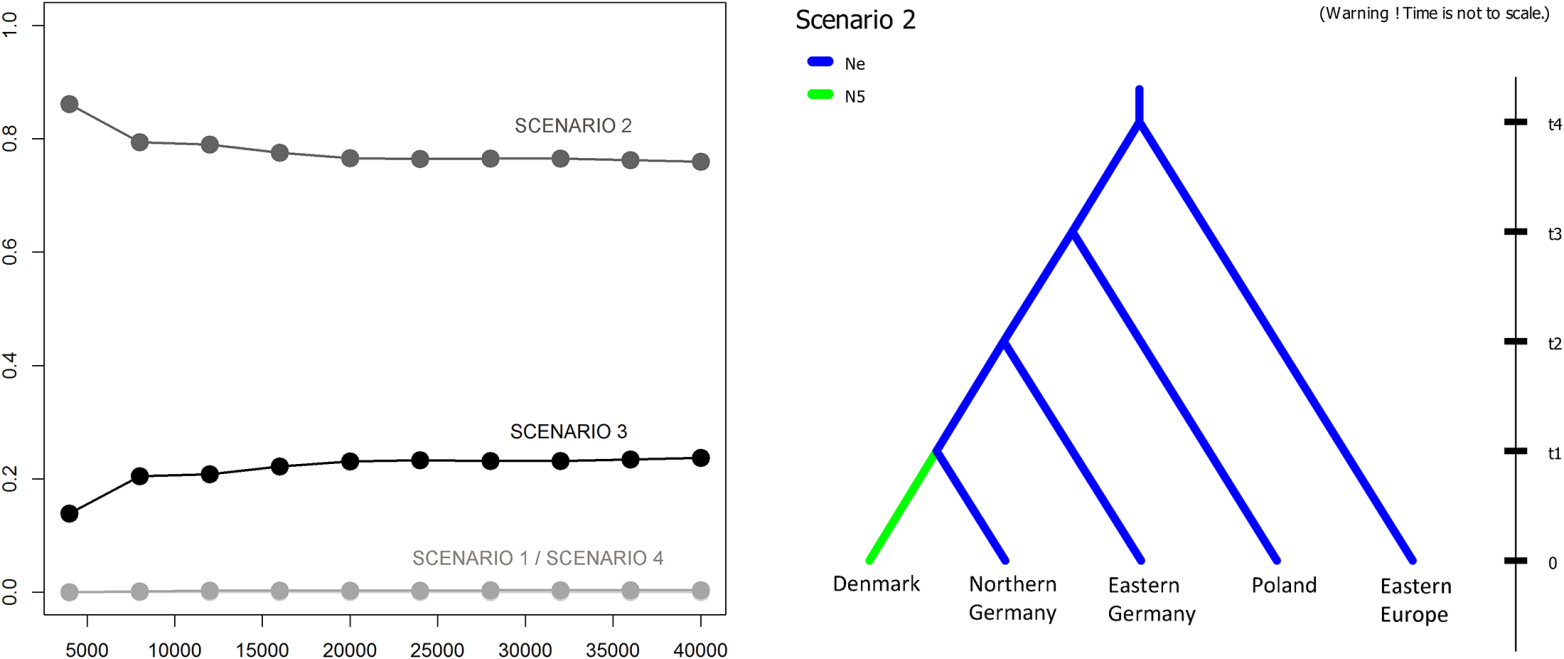
Results from the ABC analysis. Left: graph of linear regressions showing posterior probabilities of the scenarios on the Y axis and the number of simulations used to calculate it (1% of total simulations) on the X axis. Right: The plot for the best-supported scenario.

A principal component analysis demonstrated the reliability of the chosen scenarios in the DIYABC analysis. Scenario 2 was as the most probable (Fig 5 and S2 Fig), showing that, with the exception of Denmark (mode *N*_e_ = 367, *Q*_*0*.*05*_ = 166, *Q*_*0*.*95*_ = 1460), all newly founded populations had an effective populations size that was similar to the source population (mode *N*_e_ = 4440, *Q*_*0*.*05*_ = 3140, *Q*_*0*.*95*_ = 4880).

## Discussion

The clustering analyses agreed on the presence of three genetic populations in the data set. We found evidence for a large 'central population', reaching from the 'core areas' of introduction to the 'edges' of the current distribution area. The clustering, FCA, IBD and private allele analyses all suggested, however, that the Finnish animals were genetically differentiated from the central population. The presence of central cluster individuals in southern Finland and of Finnish individuals in central Europe indicates the presence of some gene flow between Finland and north-eastern Europe. The most likely explanation for the genetic distinctness therefore is that the Finnish and central European raccoon dogs descend from genetically distinct founding populations. This was already suggested by previous studies that identified one mitochondrial control region haplotype that was common in Germany and Lithuania, respectively, but absent from Finland [25, 51]. It should be noted, however, that, according to the linkage disequilibrium method, the effective population size of Finnish raccoon dogs appears to be relatively small, suggesting genetic drift might also have contributed to the genetic differentiation.

While the first raccoon dogs were observed in Finland in the 1930s, the species started to colonise the country in earnest during the mid-1950s, establishing itself in most of southern and central Finland by the mid-1970s [52]. It has therefore been suggested that the Finnish raccoon dog population descent from individuals released near Leningrad (now St. Petersburg) and in the Karelian Isthmus near Finland [9,12]. The colonisation of Poland started in earnest during the 1950s, most likely with animals originating from Belarus and the Ukraine, where 2000 raccoon dogs were released between the 1930s and the 1950s [12].

Even though mtDNA showed the presence of two distinct clades in central Europe, Russia and Lithuania [25, 51, 53], our microsatellite genotyping results indicated only one genetic cluster for samples spanning from western Russia to northern Germany. Founders were first bred in fur farms and deliberately 'soft-released' into the wild from enclosures, specifically build for this purpose [9]. Hence, this admixture of raccoon dogs from breeding programs and fur farms may have led to nuclear DNA panmixis even before the initial introduction events. Furthermore, some populations were founded by animals that had been captured in the introduction areas [12]. Because both high introduction effort and admixture of individuals of distinctly different origins may contribute to the maintenance of genetic variation [54–56], it is likely that the genetic variation of the raccoon dog in Europe is not substantially lower than in populations of the Amur and Ussuri regions of the Russian Far East where animals were originally sampled for release [9]. A study on non-metric skull characters (anomalies in the normal anatomy which reflect the genetic basis of phenotypic traits) did not find evidence for reduced phenogenetic variability, founder effects or inbreeding in European raccoon dogs [57]. In addition, although based on different microsatellite markers, overall diversity levels across genotypes of native raccoon dog populations in South Korea [58] were similar to the values presented here.

While the raccoon dogs from western Russia to northern Germany formed one genetic cluster, we did not find a significant IBD pattern between the pre-defined populations within this cluster (*P* = 0.055) and the DIYABC analysis supported a model of equal effective population sizes of all central European populations. The results reported here are therefore in line with strong gene flow and secondary admixture between neighbouring demes having reduced genetic structuring. Frequent long-distance dispersal may also have contributed to the homogenisation of the genetic structure [59], especially when considering the high population turnover reported for the species [8,60].

However, we found a tendency for genetic diversity to decrease from east to west (and in Finland to the north). Furthermore, the results of the less well-supported *K* = 4 STRUCTURE analysis suggested a weak genetic partition between the western and the eastern part of the central cluster. It is conceivable that the initial founders experienced sequential bottlenecks and that the genetic structure was homogenised as a result of recent secondary admixture, perhaps resulting from frequent long-distance dispersal events. The analysis of non-metric skull characters led to the conclusion that raccoon dogs in Germany (sampled between 1994 and 2003) formed a cluster distinct from animals sampled in both Finland an Eastern Poland [57]. In the future, we plan to genetically analyse samples collected from the same location but from different time periods to look for evidence of reduced variability and genetic distinctness of 'earlier' German raccoon dogs.

Our results showed Danish raccoon dogs to form a genetically distinct population, despite the absence of an obvious physical barrier to gene flow between Denmark and northern Germany. The DIYABC analysis showed that the Danish cluster had a smaller effective population size than the Central European cluster, probably resulting in reduced genetic diversity. It is therefore plausible that Denmark is a case in point of the scenario described above: the population was founded by just a few individuals and that founder effect will be overcome by the influx of long-distance migrants from the core population. Some BAPS runs did assign some raccoon dogs sampled in Denmark to the central European cluster. However, given the presence of microsatellite alleles that did not occur in the central European cluster, the Danish population may have been initiated by raccoon dogs that were released or escaped from commercial fur farms or pet stocks in Denmark. The first Danish raccoon dog was recorded in 1980. However, until 2008 the species was only found sporadically and some of the 25 raccoon dogs recorded between 1995 and 2003 were known to have escaped from captivity [61,62]. Since 2008, numbers have increased markedly. The development of the distribution range of raccoon dogs in Denmark strongly suggests that the population was founded by recent escapees in north-western Denmark rather than regular dispersal movements from Germany [63]. It remains to be seen whether admixture will homogenise the Danish and Central European clusters.

There are many unknowns regarding the calculation of effective population size [64]. While the LD approach is viewed as a reliable method, its point estimates are one order of magnitude lower than the estimates generated using the DIYABC approach. The different estimates therefore ought to be viewed in a comparative context: irrespective of the actual point estimates, both methods agree that the Danish raccoon dogs have a reduced effective population size compared to the central European population.

Our results are remarkable in the sense that we identified a homogenous genetic cluster inhabiting an area stretching over more than 1500km. Other invasive mammals have been shown to maintain genetic structure during introduction and similarly exhibit homogenous genetic structure covering larges spatial distances (e.g. [65,66]). The population genetic structure of native carnivores, even if they are highly mobile, is frequently [67–71], but not always [70], affected by habitat specialisation, climate, habitat barriers or simply geographic distance. In South Korea, the raccoon dog is also characterised by significant genetic structuring [58]. In Germany, the mean and maximal life-time dispersal distance of 59 marked raccoon dogs was 13.5 km and 91.2 km respectively [72]. Theory has shown that the rate of dispersal of individual animals and plants should increase towards the front of an expanding geographic range [73, 74]. In other words, the homogenous population genetic structure observed in the raccoon dog in Europe is probably a result of its fairly rapid population expansion after introduction.

The results presented here have great relevance for disease management. First, the extent of its genetic homogeneity suggests the lack of any substantial landscape barrier to dispersal. Secondly, the absence of a (strong) IBD pattern, as well as some direct evidence from our assignment results, suggests frequent long-distance dispersal. This is in line with the speed of the historic spread of the species, as well as with several field studies reporting raccoon dog dispersal over large distances in relatively short period of time (e.g. [72,75,76]), particularly as a result of long-distance dispersal of young raccoon dogs [19]. Thus, in the event of a significant rabies outbreak, there is a great risk of a rapid virus spread among raccoon dog populations. While comparison with a study from South Korea [58] suggested that European raccoon dogs were not genetically depauperate, the species was both main vector and victim during a rabies epizootic in Finland at the end of the 1980s, [77,78].

At present, it is impossible to know whether a pattern of high population connectivity and frequent long-distance dispersal, together with the resulting homogenous genetic structure, will be maintained after the end of the species spatial expansion. While perhaps species-specific, there are examples in the literature of reduced dispersal in high-density populations [79]. Because it is impossible to know the future, managers should be aware of the species potential to spread diseases over long distances when designing effective management and vaccination strategies. If not already put in practice, countries should systematically screen raccoon dog carcasses for diseases, especially in the newly colonised areas in Western Europe.

## Supporting Information

S1 Fig Effective geographic distance used in the calculation of the isolation-by-distance pattern involving the pre-defined populations. The red star indicates the geographic coordinate for each pre-defined population (the average longitude and latitude of the individual samples in a pre-defined population) and the black lines the least-cost distance separating the populations (obtained using a resistance surface with a high cost value for water bodies).
(TIF)

S2 Fig Graphical representation of the four competing phylogeographic scenarios considered in the DIYABC analysis. Pop 1 = Source population (Russia, Estonia, Lithuania), Pop 2 = Eastern Poland; Pop 3 = Brandenburg/Saxony; Pop 4 = Schleswig-Holstein; Pop 5 = Denmark.
(TIF)

S3 Fig Inference of genetic clusters using the STRUCTURE algorithm. Plot of the number of genetic clusters tested against their estimated log-likelihood. STRUCTURE was run using the admixture and correlated allele frequencies models.
(TIFF)

S1 Table Properties of the microsatellite multiplex panel used in this study. (DOCX)

S2 Table Significance values of GENEPOP exact test for Hardy-Weinberg deviations. (DOCX)

S3 Table Genetic characteristics of the 10 pre-defined populations. (DOCX)
