# Supplementary figures and images for: Homogenous Population Genetic Structure of the Non-Native Raccoon Dog (Nyctereutes procyonoides) in Europe as a Result of Rapid Population Expansion
Source: PLoS One. 2016 Apr 11;11(4):e0153098. doi: 10.1371/journal.pone.0153098 (PMC4827816; doi:10.1371/journal.pone.0153098)

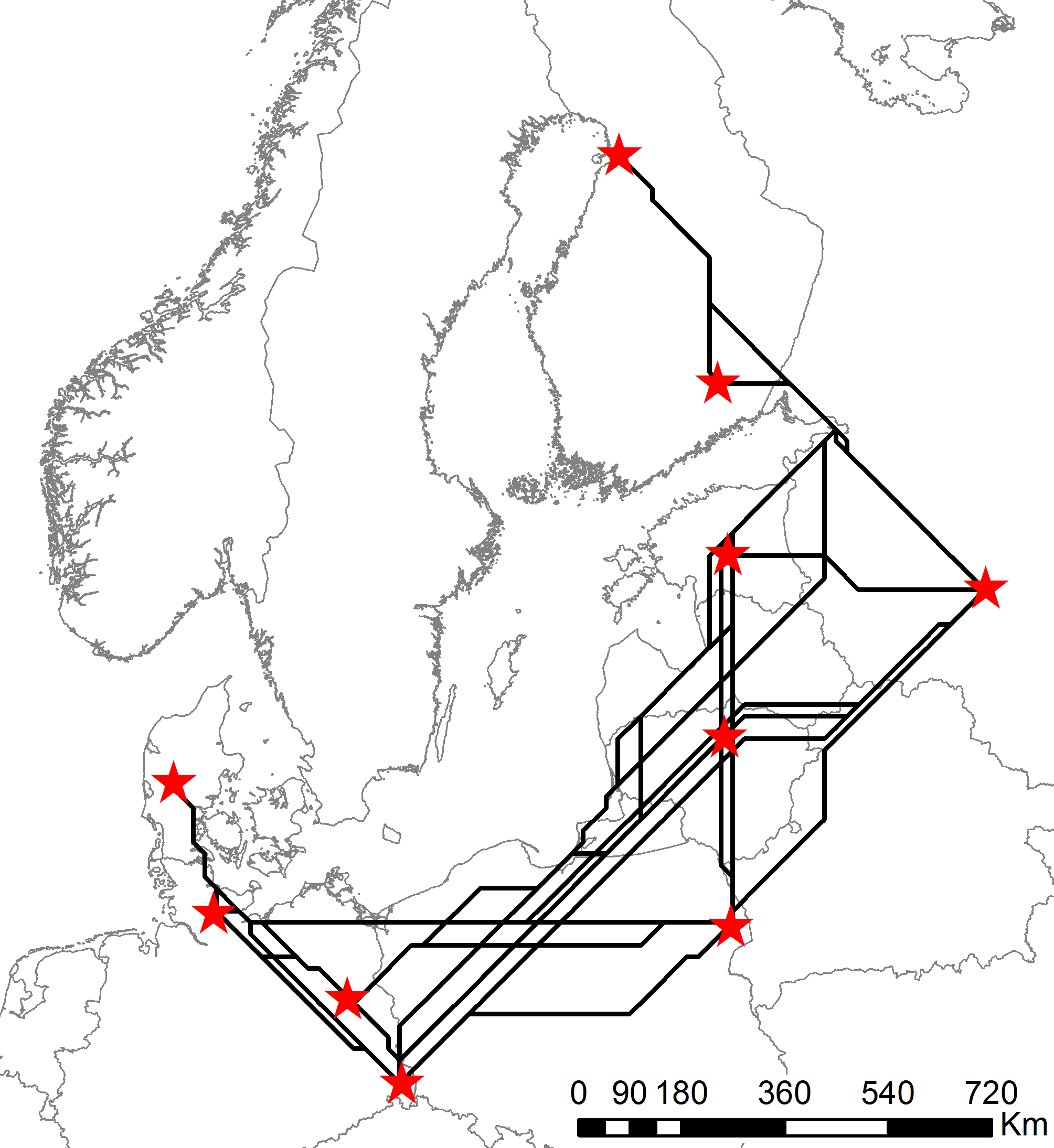

Supplement: S1 Fig — The red star indicates the geographic coordinate for each pre-defined population (the average longitude and latitude of the individual samples in a pre-defined population) and the black lines the least-cost distance separating the populations (obtained using a resistance surface with a high cost value for water bodies). (TIF) [file pone.0153098.s001.tif]

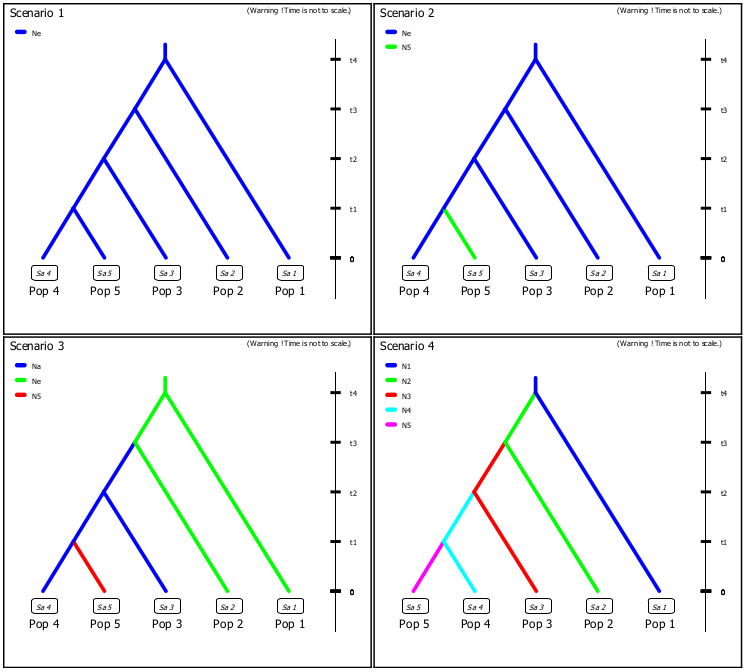

Supplement: S2 Fig — Pop 1 = Source population (Russia, Estonia, Lithuania), Pop 2 = Eastern Poland; Pop 3 = Brandenburg/Saxony; Pop 4 = Schleswig-Holstein; Pop 5 = Denmark. (TIF) [file pone.0153098.s002.tif]

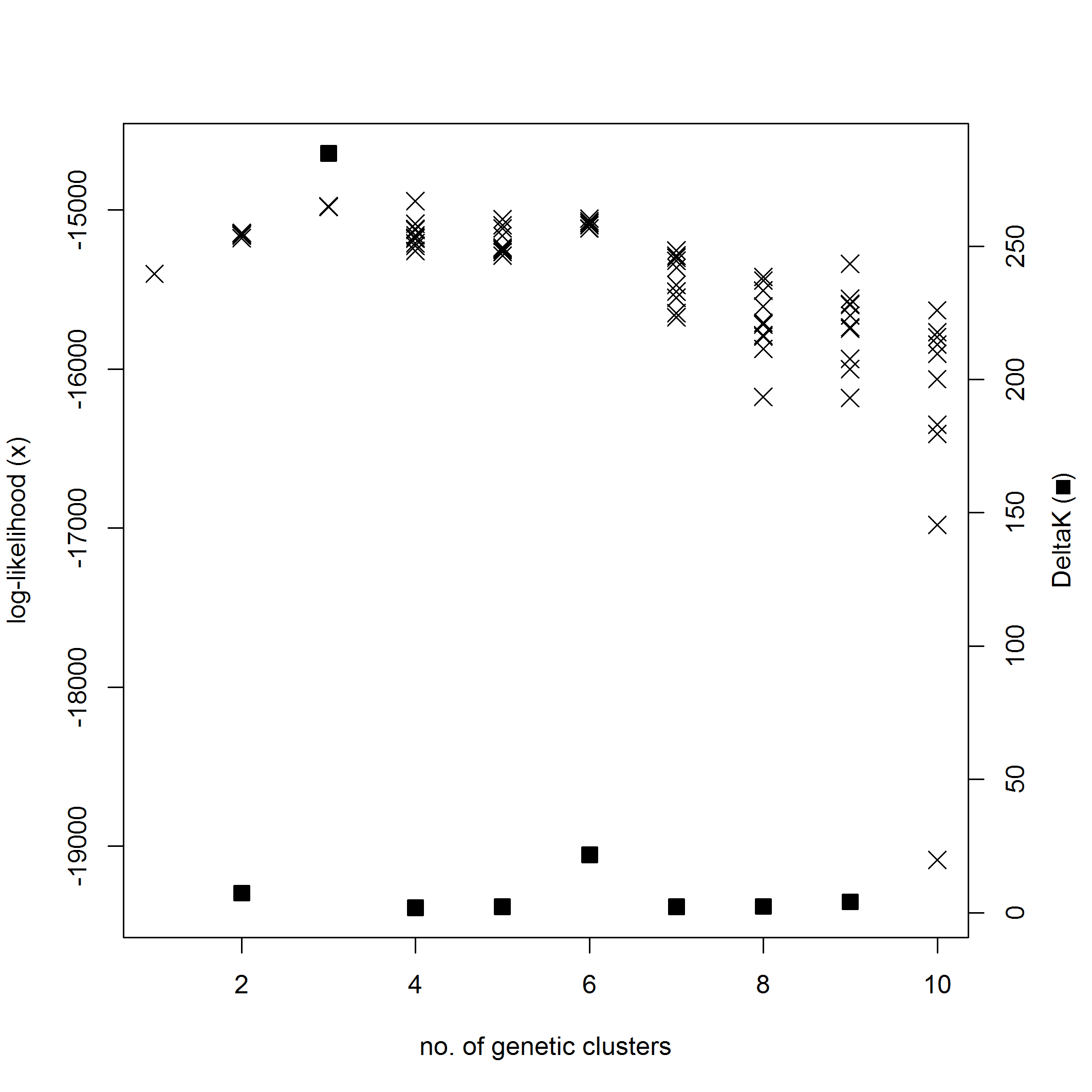

Supplement: S3 Fig — Plot of the number of genetic clusters tested against their estimated log-likelihood. STRUCTURE was run using the admixture and correlated allele frequencies models. (TIFF) [file pone.0153098.s003.tiff]
