# Supplementary material for: Homogenous Population Genetic Structure of the Non-Native Raccoon Dog (Nyctereutes procyonoides) in Europe as a Result of Rapid Population Expansion
Source: PLoS One. 2016 Apr 11;11(4):e0153098. doi: 10.1371/journal.pone.0153098 (PMC4827816; doi:10.1371/journal.pone.0153098)
Supplement: S1 Table — (DOCX) [file pone.0153098.s004.docx]

S1 Table. Properties of the microsatellite multiplex panel used in this study. System = electrophoresis loading multiplex; Primer (μM) = final primer concentration in μM; *N* = number of samples analysed successfully; *A* = number of alleles.

|  | System | Dye | Primer (μM) | Allele range (bp) | *N* | *A* |
| --- | --- | --- | --- | --- | --- | --- |
| **Multiplex 1** |  |  |  |  |  |  |
| *DGN14* | 1 | 6-FAM | 0.2 | 248-314 | 328 | 23 |
| *RenB09* | 1 | HEX | 0.1 | 191-197 | 322 | 4 |
| *V142* | 1 | HEX | 0.1 | 109-130 | 330 | 8 |
| *V402* | 1 | 6-FAM | 0.2 | 73-90 | 326 | 6 |
| **Multiplex 2** |  |  |  |  |  |  |
| *FH2226* | 1 | 6-FAM | 0.2 | 144-186 | 319 | 11 |
| *FH2281* | 1 | 6-FAM | 0.3 | 392-413 | 322 | 5 |
| **Multiplex 3** |  |  |  |  |  |  |
| *FH2097* | 2 | 6-FAM | 0.2 | 263-293 | 331 | 8 |
| *FH2289* | 2 | HEX | 0.1 | 202-219 | 331 | 4 |
| *FH2316* | 2 | HEX | 0.2 | 280-350 | 327 | 19 |
| *FH2541* | 2 | HEX | 0.1 | 151-179 | 332 | 8 |
| **Multiplex 4** |  |  |  |  |  |  |
| *FH2174* | 3 | HEX | 0.1 | 166-190 | 322 | 7 |
| *FH2658* | 3 | 6-FAM | 0.2 | 302-394 | 303 | 29 |
| *RenA12* | 3 | HEX | 0.1 | 280-296 | 326 | 8 |
| *V374* | 3 | HEX | 0.1 | 95-97 | 325 | 2 |
| *V602* | 3 | 6-FAM | 0.2 | 128-154 | 329 | 9 |
