# Supplementary material for: Homogenous Population Genetic Structure of the Non-Native Raccoon Dog (Nyctereutes procyonoides) in Europe as a Result of Rapid Population Expansion
Source: PLoS One. 2016 Apr 11;11(4):e0153098. doi: 10.1371/journal.pone.0153098 (PMC4827816; doi:10.1371/journal.pone.0153098)
Supplement: S2 Table — (DOCX) [file pone.0153098.s005.docx]

S2 Table. Validity of the 15 microsatellite markers for population genetic analyses (a) Significance values of GENEPOP exact test for Hardy-Weinberg deviations. Values in bold are significant before correction for multiple tests, the values marked with an asterisk are significant after this correction. (b) Estimates of null allele frequencies obtained using FreeNA. Estimated null allele frequencies of >0.10 are in bold

(a)

| Population | Microsatellite locus | | | | | | | | | | | | | | | |
| --- | --- | --- | --- | --- | --- | --- | --- | --- | --- | --- | --- | --- | --- | --- | --- | --- |
|  | DGN14 | FH2097 | FH2174 | FH2226 | FH2281 | FH2289 | FH2316 | FH2541 | FH2658 | RenA12 | RenB09 | V142 | V374 | V402 | V468 | V602 |
| Denmark | 0.840 | 0.602 | **0.004** | 0.123 | 1.000 | 0.588 | 0.235 | 0.350 | **0.045** | 0.325 | **0.033** | 0.430 | 0.703 | 0.678 | 0.893 | 0.692 |
| Brandenburg | 0.749 | 0.570 | **<0.001*** | 0.886 | 0.503 | 0.696 | 0.487 | 0.844 | 0.443 | 0.719 | 0.556 | 0.602 | 0.274 | 0.594 | 0.538 | 0.785 |
| Estonia | 0.111 | 0.766 | **<0.001*** | **0.038** | 1.000 | 0.736 | 0.906 | 0.603 | 0.166 | 0.806 | 0.850 | 0.167 | 0.225 | 0.159 | 0.331 | 0.673 |
| Finland Central | 0.201 | 0.079 | 0.103 | **0.012** | 1.000 | 0.198 | 0.613 | **0.007** | 0.498 | 0.326 | 1.000 | **0.009** | 0.662 | 0.371 | **0.028** | 0.667 |
| Finland South | **0.001*** | 0.860 | **0.003** | 0.633 | 0.381 | 0.970 | 0.120 | 0.517 | 0.257 | 0.189 | 0.393 | 0.941 | 0.725 | 0.149 | 0.427 | 0.613 |
| Lithuania | 0.471 | 0.971 | **<0.001*** | 0.251 | 0.112 | 0.073 | 0.164 | 0.608 | 0.228 | 0.093 | 0.425 | 0.710 | 1.000 | 0.457 | 0.747 | 0.096 |
| Poland East | 0.371 | 0.464 | **<0.001*** | **0.003*** | 0.663 | 0.659 | **0.001*** | 0.218 | **0.045** | 0.783 | 0.226 | 0.184 | 0.774 | 0.108 | 0.974 | 0.495 |
| Russia | 0.182 | 0.621 | **<0.001*** | 0.776 | 1.000 | 0.514 | 0.427 | 0.360 | 0.599 | 0.266 | 0.139 | **0.013** | 1.000 | 0.641 | 0.126 | 0.920 |
| Saxony | 0.872 | 0.638 | **0.007** | **0.024** | 1.000 | 0.785 | 0.620 | 0.473 | 0.562 | 0.841 | 0.512 | 0.897 | 0.368 | 0.239 | 0.209 | 0.414 |
| Schleswig-Holstein | 0.810 | 0.711 | **<0.001*** | 0.633 | 0.153 | 1.000 | 0.370 | 0.408 | 0.576 | 0.576 | 1.000 | 0.203 | 0.218 | 0.938 | 0.748 | 0.175 |

(b)

| Population | Microsatellite locus | | | | | | | | | | | | | | | |
| --- | --- | --- | --- | --- | --- | --- | --- | --- | --- | --- | --- | --- | --- | --- | --- | --- |
|  | DGN14 | FH2097 | FH2174 | FH2226 | FH2281 | FH2289 | FH2316 | FH2541 | FH2658 | RenA12 | RenB09 | V142 | V374 | V402 | V468 | V602 |
| Denmark | 0.000 | 0.000 | **0.129** | 0.000 | 0.000 | 0.000 | 0.000 | 0.000 | 0.000 | 0.000 | 0.000 | 0.000 | 0.000 | 0.000 | 0.009 | 0.000 |
| Brandenburg | 0.000 | 0.000 | **0.208** | 0.000 | 0.019 | 0.015 | 0.009 | 0.000 | 0.000 | 0.037 | 0.000 | 0.000 | 0.000 | 0.000 | 0.016 | 0.000 |
| Estonia | 0.000 | 0.052 | **0.170** | 0.033 | 0.000 | 0.000 | 0.000 | 0.063 | 0.045 | 0.000 | 0.000 | 0.000 | 0.078 | 0.000 | 0.012 | 0.056 |
| Finland Central | 0.000 | 0.051 | 0.096 | 0.022 | 0.000 | 0.000 | 0.000 | 0.043 | 0.000 | 0.000 | 0.000 | 0.077 | 0.000 | 0.023 | **0.121** | 0.001 |
| Finland South | 0.060 | 0.001 | **0.104** | 0.000 | 0.043 | 0.000 | 0.052 | 0.000 | 0.007 | 0.000 | 0.047 | 0.000 | 0.026 | 0.000 | 0.000 | 0.000 |
| Lithuania | 0.000 | 0.000 | **0.219** | 0.010 | 0.000 | 0.072 | 0.000 | 0.010 | 0.000 | 0.059 | 0.000 | 0.032 | 0.000 | 0.000 | 0.008 | 0.087 |
| Poland East | 0.040 | 0.040 | **0.280** | 0.059 | 0.000 | 0.000 | 0.034 | 0.000 | 0.080 | 0.000 | 0.006 | 0.018 | 0.020 | 0.052 | 0.000 | 0.018 |
| Russia | 0.045 | 0.000 | **0.280** | 0.000 | 0.000 | 0.042 | 0.000 | 0.010 | 0.012 | 0.055 | 0.039 | 0.010 | 0.013 | 0.022 | 0.000 | 0.000 |
| Saxony | 0.013 | 0.023 | **0.180** | 0.017 | 0.000 | 0.031 | 0.000 | 0.000 | 0.000 | 0.000 | 0.000 | 0.000 | 0.000 | 0.000 | **0.115** | 0.000 |
| Schleswig-Holstein | 0.013 | 0.027 | **0.206** | 0.000 | 0.079 | 0.000 | 0.000 | 0.000 | 0.000 | 0.000 | 0.000 | 0.000 | 0.093 | 0.003 | 0.000 | 0.025 |
