# Supplementary material for: Homogenous Population Genetic Structure of the Non-Native Raccoon Dog (Nyctereutes procyonoides) in Europe as a Result of Rapid Population Expansion
Source: PLoS One. 2016 Apr 11;11(4):e0153098. doi: 10.1371/journal.pone.0153098 (PMC4827816; doi:10.1371/journal.pone.0153098)
Supplement: S3 Table — (DOCX) [file pone.0153098.s006.docx]

S3 Table. Genetic characteristics of the 10 pre-defined populations (*A*: no. of alleles; *A*_R_: allelic richness (based on a minimum sample size of 19 diploid individuals); *Ho*: observed heterozygosity; *He*_u_: unbiased expected heterozygosity; *N*_e_: effective population size estimated using the linkage disequilibrium method.

| Country | Region within country | Sample size | | Microsatellite diversity | | | | *N*_e_ | 95% CI |
| --- | --- | --- | --- | --- | --- | --- | --- | --- | --- |
|  |  |  |  | *A* | *A*_R_ | *Ho* | *He*_u_ |  |  |
| Russia | West | 27 |  | 6.9 | 6.5 | 0.656 | 0.679 | 161.4 | 60.5-infinite |
| Lithuania |  | 36 |  | 7.3 | 6.5 | 0.693 | 0.710 | 57.5 | 44.3-79.4 |
| Estonia |  | 31 |  | 7.1 | 6.4 | 0.678 | 0.696 | 286.7 | 96.9- infinite |
| Finland | South | 34 |  | 6.9 | 6.1 | 0.656 | 0.667 | 384.8 | 117.6-infinite |
| Finland | Centre | 23 |  | 5.9 | 5.6 | 0.650 | 0.672 | 26.4 | 19.6-38.0 |
| Poland | East | 51 |  | 7.4 | 6.2 | 0.686 | 0.705 | 100.7 | 74.4-149.3 |
| Germany | Saxony | 20 |  | 5.7 | 5.7 | 0.689 | 0.678 | 97.1 | 47.7-1350.7 |
| Germany | Brandenburg | 55 |  | 7.0 | 5.8 | 0.677 | 0.680 | 415 | 128.3-infinite |
| Germany | Schleswig-Holstein | 26 |  | 5.7 | 5.4 | 0.684 | 0.672 | 59 | 34.5-154.7 |
| Denmark |  | 29 |  | 5.1 | 4.7 | 0.658 | 0.607 | 35.1 | 22.1-68.9 |
